# Supplementary material for: Dopamine lesions alter the striatal encoding of single-limb gait
Source: bioRxiv. 2024 Jan 11:2023.10.06.561216. Originally published 2023 Oct 9. Preprint. [Version 2] doi: 10.1101/2023.10.06.561216 (PMC10592622; doi:10.1101/2023.10.06.561216)
Supplement: Supplement 1 [file NIHPP2023.10.06.561216v2-supplement-1.pdf]

bioRxiv preprint doi: https://doi.org/10.1101/2023.10.06.561216; this version posted January 11, 2024. The copyright holder for this preprint (which was not certified by peer review) is the author/funder, who has granted bioRxiv a license to display the preprint in perpetuity. It is made available under aCC-BY 4.0 International license.

FIGURE SUPPLEMENTS

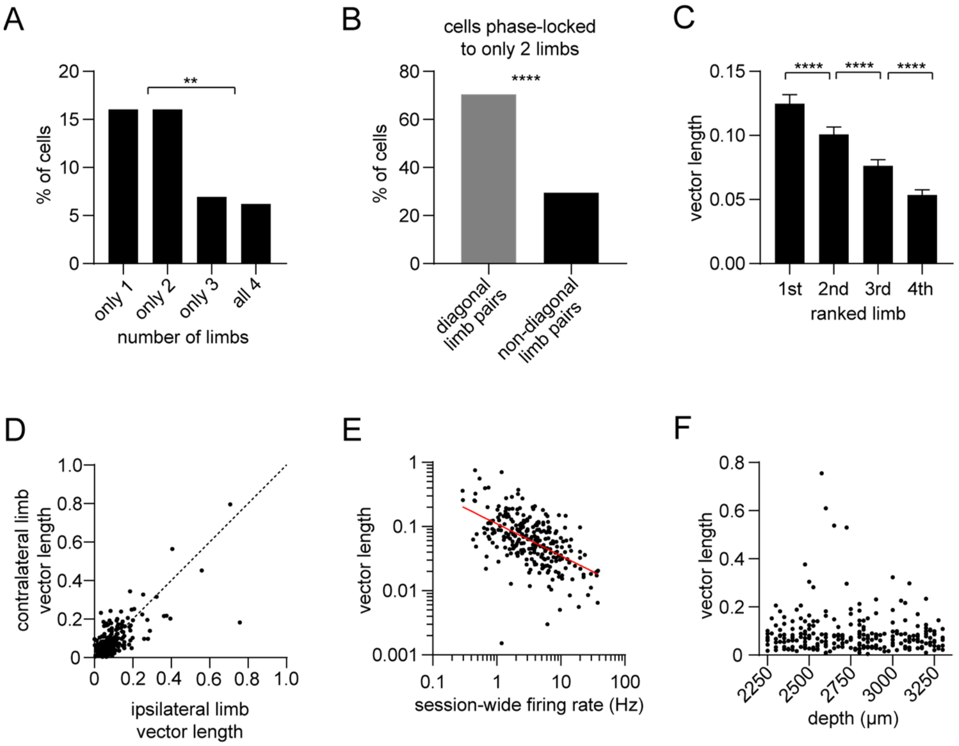

Figure 2 – Figure Supplement 1. Single-limb phase locking strength in dorsal striatal neurons.

(A) Percentage of striatal neurons with significant phase-locking to only 1, only 2, only 3, or all 4 limbs (n = 274 total cells pooled from 9 healthy mice, chi-square test adjusted for 6 comparisons, 1 vs 3 and 2 vs 3: p = 0.003; 1 vs 4 and 2 vs 4: p = 0.0009).

(B) Among the neurons which were phase-locked to only 2 limbs (n = 44 cells), a greater proportion of limb pairs were diagonal (chi-square test, p < 0.0001).

(C) Significant difference in spike-limb phase vector length when averaged by limb rank in order of highest to lowest vector length (n = 274 cells, one-way RM ANOVA, p < 0.0001). Data represent mean ± SEM.

(D) The mean vector length of striatal neurons shows no preference for the contralateral (LF) or ipsilateral (RF) limb (paired t-test, p = 0.3). Each dot represents one neuron.

(E) Significant negative correlation between the vector length and session-wide firing rate per cell. Vector length is calculated from the LF limb (n = 274 cells, Pearson r = -0.32, p < 0.0001). Each dot represents one neuron. Red line represents the best linear fit, plotted on a logarithmic scale.

(F) Mean vector length per cell as a function of the neuron's estimated depth in the dorsal striatum relative to bregma (Pearson r = -0.08, p = 0.22). Each dot represents one neuron.

bioRxiv preprint doi: https://doi.org/10.1101/2023.10.06.561216; this version posted January 11, 2024. The copyright holder for this preprint (which was not certified by peer review) is the author/funder, who has granted bioRxiv a license to display the preprint in perpetuity. It is made available under aCC-BY 4.0 International license.

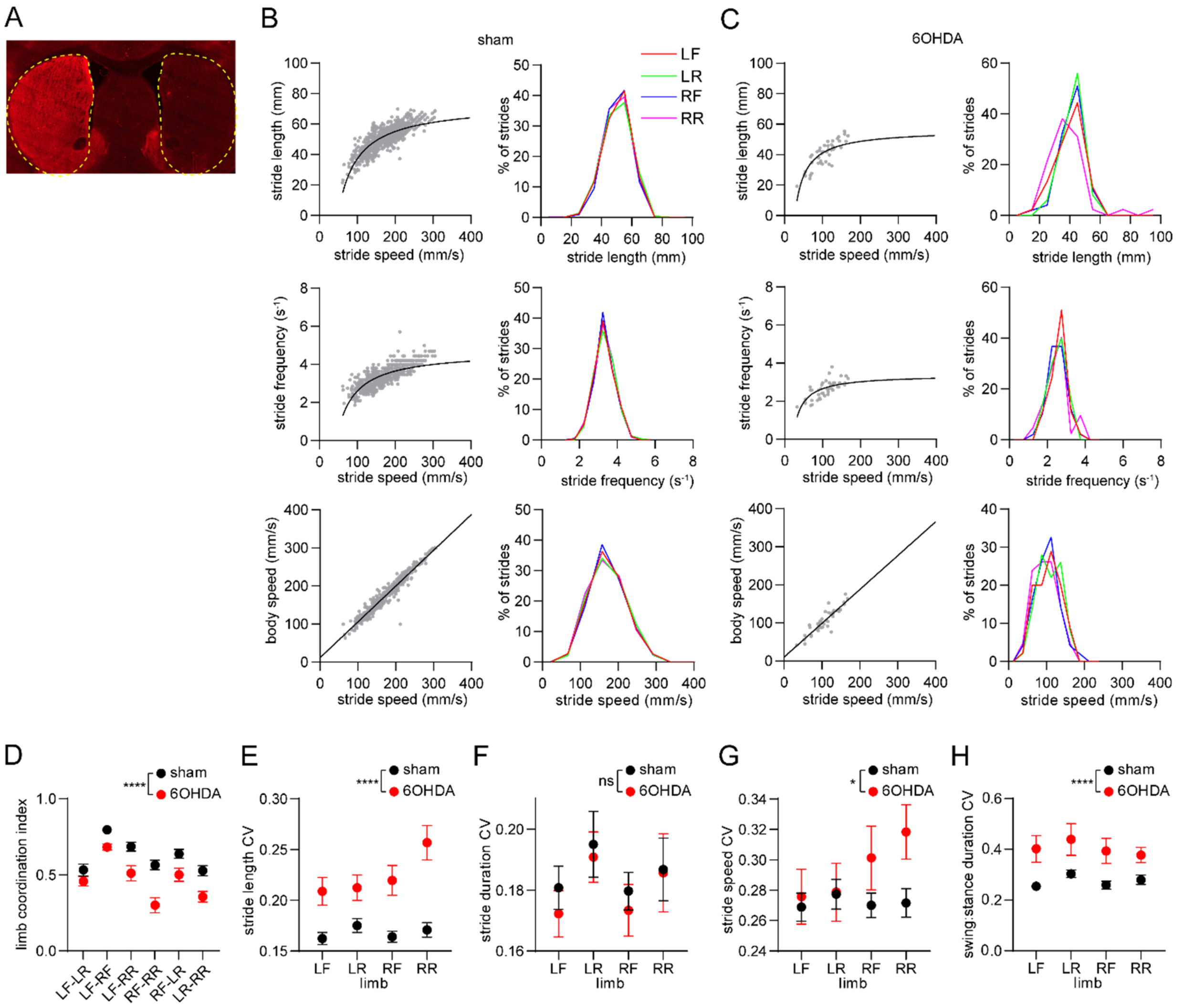

Figure 5 – Figure Supplement 1. Limb coordination and gait variability are altered by dopamine lesions.

(A) Fluorescence image of a brain section from a 6OHDA-lesioned mouse, immunostained for tyrosine hydroxylase. Dashed yellow lines demarcate the striatum.

(B) Limb stride parameters from one recording session in the sham group. Gray dots represent individual LF limb strides. Black lines represent the best polynomial or linear fit.

(C) Limb stride parameters from one recording session in the 6OHDA group.

(D) The coordination between different limb pairs is significantly reduced in the 6OHDA group (n = 10 6OHDA and 14 sham-lesioned mice, two-way ANOVA, $F_{1,132} = 61$, $p < 0.0001$).

(E) Mean coefficient of variation (CV) in stride length is significantly increased in the 6OHDA group (two-way ANOVA, $F_{1,88} = 61$, $p < 0.0001$).

(F) Mean CV in stride duration does not significantly change in 6OHDA lesioned mice (two-way ANOVA, $F_{1,88} = 0.6$, $p = 0.45$).

(G) Mean CV in stride speed is significantly increased in the 6OHDA group (two-way ANOVA, $F_{1,88} = 5$, $p = 0.03$).

(H) Mean CV in the stride swing:stance duration is significantly increased in the 6OHDA group (two-way ANOVA, $F_{1,88} = 31$, $p < 0.0001$). All data are represented as mean ± SEM.

bioRxiv preprint doi: https://doi.org/10.1101/2023.10.06.561216; this version posted January 11, 2024. The copyright holder for this preprint (which was not certified by peer review) is the author/funder, who has granted bioRxiv a license to display the preprint in perpetuity. It is made available under aCC-BY 4.0 International license.

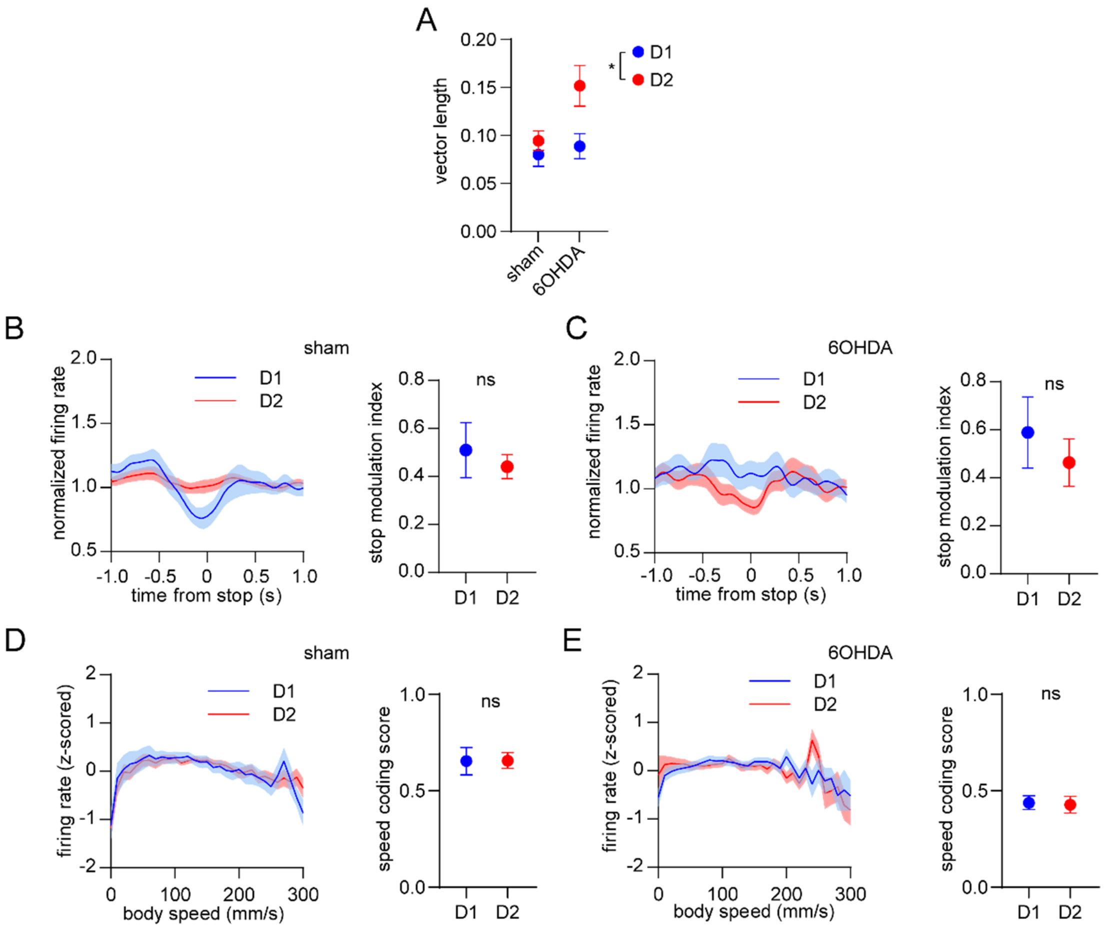

Figure 6 – Figure Supplement 1. Dopamine lesions do not alter the balanced D1/D2 MSN encoding of movement cessation and body speed.

(A) Cell type (D1 or D2 MSN), group (sham or 6OHDA) and the interaction between them have a significant effect on the average vector length across the four limbs (n = 22 D1 and 57 D2 MSNs in the sham group, 31 D1 and 28 D2 MSNs in the 6OHDA group, two-way ANOVA, cell type factor: $F_{1,134} = 7$, p = 0.01, group factor: $F_{1,134} = 5$, p = 0.03, interaction: $F_{1,134} = 3$, p = 0.1).

(B) Left: normalized firing rate relative to the cessation of movement averaged across all D1 and D2 MSNs in the sham group. Data are normalized to the mean firing rate in a pre-stop period. Right: No significant difference in the stop modulation index (fractional change in firing in stop period relative to pre-stop) between D1 and D2 MSNs (n = 22 D1 and 57 D2 MSNs, unpaired t-test, p = 0.52).

(C) Left: normalized firing rate relative to the cessation of movement averaged across all D1 and D2 MSNs in the 6OHDA group. Data are normalized to the mean firing rate in a pre-stop period. Right: No significant difference in the stop modulation index between D1 and D2 MSNs (n = 31 D1 and 28 D2 MSNs, unpaired t-test, p = 0.5).

(D) Left: firing rate (z-scored) as a function of body speed averaged across all D1 and D2 MSNs in the sham group. Right: No significant difference in speed coding score (absolute Pearson r of firing rate in relation to speed) between D1 and D2 MSNs (n = 22 D1 and 57 D2 MSNs, unpaired t-test, p = 0.97).

(E) Left: firing rate (z-scored) as a function of body speed averaged across all D1 and D2 MSNs in the 6OHDA group. Right: No significant difference in speed coding score between D1 and D2 MSNs (n = 31 D1 and 28 D2 MSNs, unpaired t-test, p = 0.93). All data are represented as mean ± SEM.

bioRxiv preprint doi: https://doi.org/10.1101/2023.10.06.561216; this version posted January 11, 2024. The copyright holder for this preprint (which was not certified by peer review) is the author/funder, who has granted bioRxiv a license to display the preprint in perpetuity. It is made available under aCC-BY 4.0 International license.

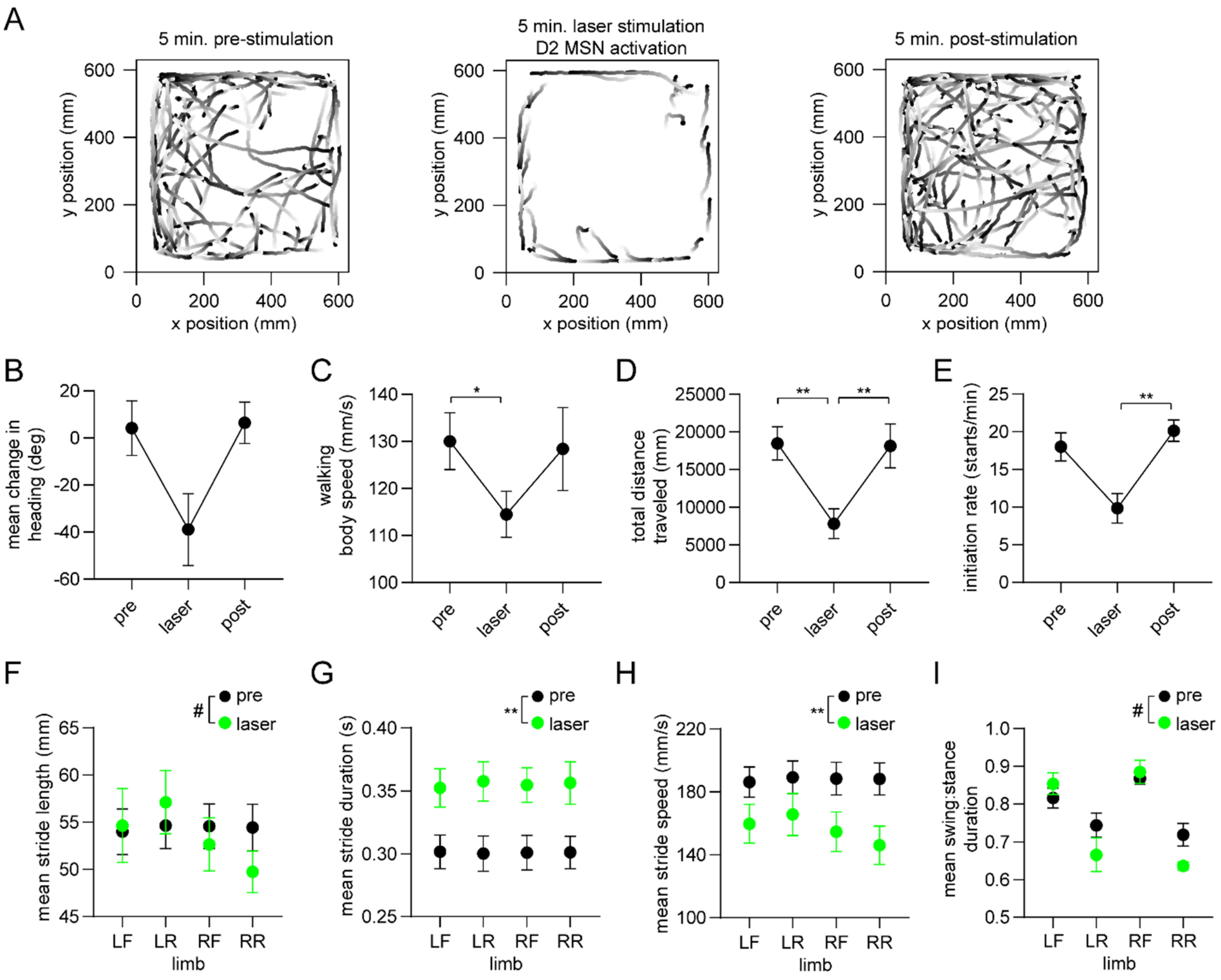

Figure 6 – Figure Supplement 2. Optogenetic activation of D2 MSNs alters whole-body movement and single-limb gait.

(A) Walking bout body trajectories from one 15 minute recording session during which the middle 5 minutes coincided with continuous optogenetic activation of striatal D2 MSNs in the right hemisphere. Light color represents the start of movement.

(B) Mean change in movement direction in the pre-stimulation, laser, and post-stimulation periods. Negative angular values indicate ipsiversive turning (n = 4 mice, angular permutation test adjusted for 3 comparisons, $p > 0.05$).

(C) Mean body speed in the pre-stimulation, laser, and post-stimulation periods (n = 4 mice, one-way RM ANOVA, $p = 0.01$).

(D) Total distance covered in the pre-stimulation, laser, and post-stimulation periods (n = 4 mice, one-way RM ANOVA, $p < 0.0001$).

(E) Rate of initiating movements in the pre-stimulation, laser, and post-stimulation periods (n = 4 mice, one-way RM ANOVA, $p = 0.03$).

(F) Mean stride length per limb in the pre-stimulation and laser periods (n = 4 mice, two-way ANOVA, pre vs laser: $F_{1,3} = 0.9$, $p = 0.42$. Post-hoc multiple comparison tests revealed a significant difference for the RR limb: $^{\#}p = 0.03$).

(G) Mean stride duration per limb in the pre-stimulation and laser periods (n = 4 mice, two-way ANOVA, pre vs laser: $F_{1,3} = 83$, $p = 0.003$).

bioRxiv preprint doi: https://doi.org/10.1101/2023.10.06.561216; this version posted January 11, 2024. The copyright holder for this preprint (which was not certified by peer review) is the author/funder, who has granted bioRxiv a license to display the preprint in perpetuity. It is made available under aCC-BY 4.0 International license.

(H) Mean stride speed per limb in the pre-stimulation and laser periods (n = 4 mice, two-way ANOVA, pre vs laser: $F_{1,3}$ = 161, p = 0.001).

(I) Mean stride swing:stance ratio per limb in the pre-stimulation and laser periods (n = 4 mice, two-way ANOVA, pre vs laser: $F_{1,3}$ = 1.7, p = 0.28. Post-hoc multiple comparison tests revealed a significant difference for the LR and RR limbs: $^{\#}$p = 0.02).
