# Supplementary material for: Dopamine lesions alter the striatal encoding of single-limb gait
Source: bioRxiv. 2023 Oct 9:2023.10.06.561216. Preprint. [Version 1] doi: 10.1101/2023.10.06.561216 (PMC10592622; doi:10.1101/2023.10.06.561216)
Supplement: Supplementary file 1 [file NIHPP2023.10.06.561216v1-supplement-1.pdf]

bioRxiv preprint doi: https://doi.org/10.1101/2023.10.06.561216; this version posted October 9, 2023. The copyright holder for this preprint (which was not certified by peer review) is the author/funder, who has granted bioRxiv a license to display the preprint in perpetuity. It is made available under aCC-BY 4.0 International license.

SUPPLEMENTARY FIGURES

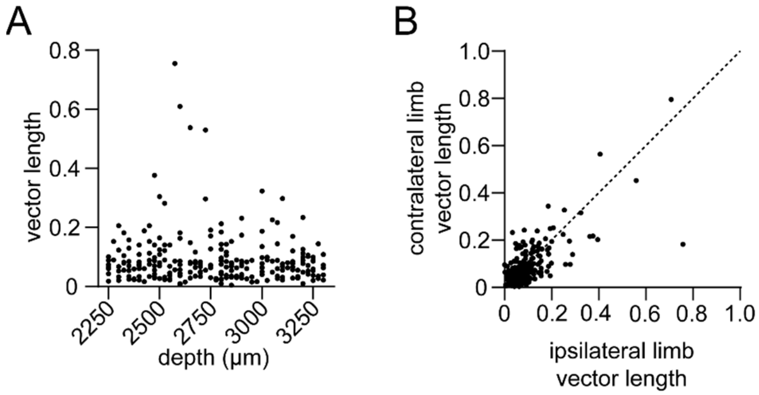

Figure S1. Single-limb phase locking strength in dorsal striatal neurons show no spatial or limb preference.

(A) Mean vector length per cell as a function of the neuron's estimated depth in the dorsal striatum relative to bregma (Pearson r = -0.08, p = 0.22). Each dot represents one neuron.

(B) The mean vector length of striatal neurons shows no preference for the contralateral (LF) or ipsilateral (RF) limb (paired t-test, p = 0.3). Each dot represents one neuron.

bioRxiv preprint doi: https://doi.org/10.1101/2023.10.06.561216; this version posted October 9, 2023. The copyright holder for this preprint (which was not certified by peer review) is the author/funder, who has granted bioRxiv a license to display the preprint in perpetuity. It is made available under aCC-BY 4.0 International license.

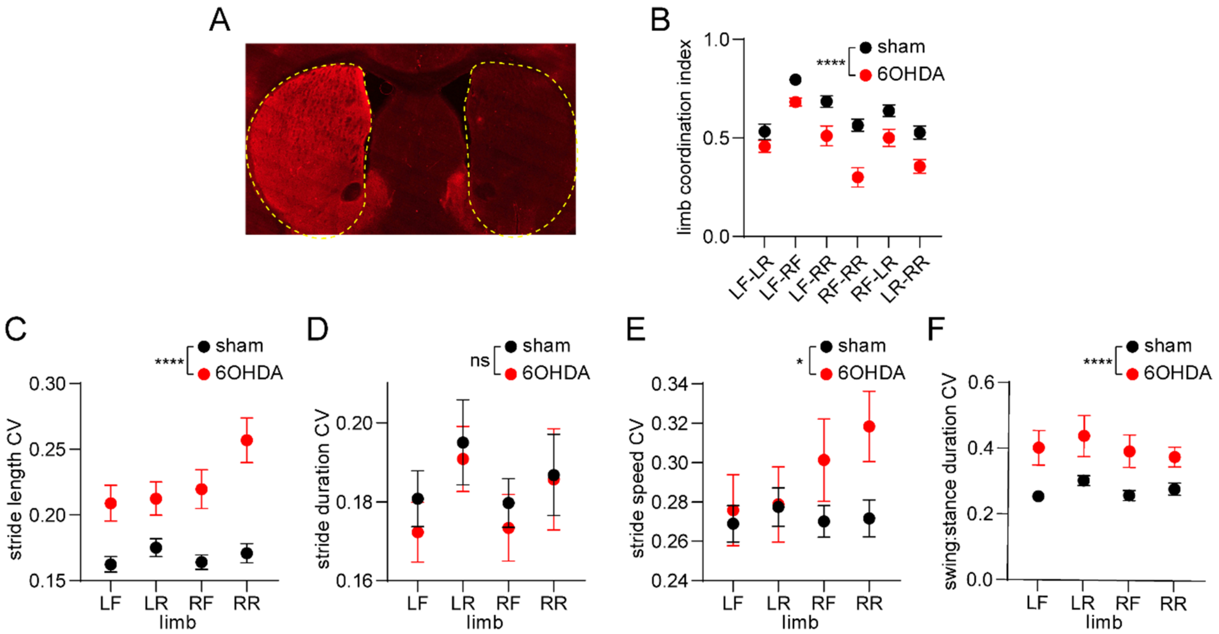

Figure S2. Limb coordination and gait variability are altered by dopamine lesions.
(A) Fluorescence image of a brain section from a 6OHDA-lesioned mouse, immunostained for tyrosine hydroxylase. Dashed yellow lines demarcate the striatum.
(B) The coordination between different limb pairs is significantly reduced in the 6OHDA group (n = 10 6OHDA and 14 sham-lesioned mice, two-way ANOVA, $F_{1,132} = 61$, $p < 0.0001$).
(C) Mean coefficient of variation (CV) in stride length is significantly increased in the 6OHDA group (two-way ANOVA, $F_{1,88} = 61$, $p < 0.0001$).
(D) Mean CV in stride duration does not significantly change in 6OHDA lesioned mice (two-way ANOVA, $F_{1,88} = 0.6$, $p = 0.45$).
(E) Mean CV in stride speed is significantly increased in the 6OHDA group (two-way ANOVA, $F_{1,88} = 5$, $p = 0.03$).
(F) Mean CV in the stride swing:stance duration is significantly increased in the 6OHDA group (two-way ANOVA, $F_{1,88} = 31$, $p < 0.0001$). All data are represented as mean ± SEM.

bioRxiv preprint doi: https://doi.org/10.1101/2023.10.06.561216; this version posted October 9, 2023. The copyright holder for this preprint (which was not certified by peer review) is the author/funder, who has granted bioRxiv a license to display the preprint in perpetuity. It is made available under aCC-BY 4.0 International license.

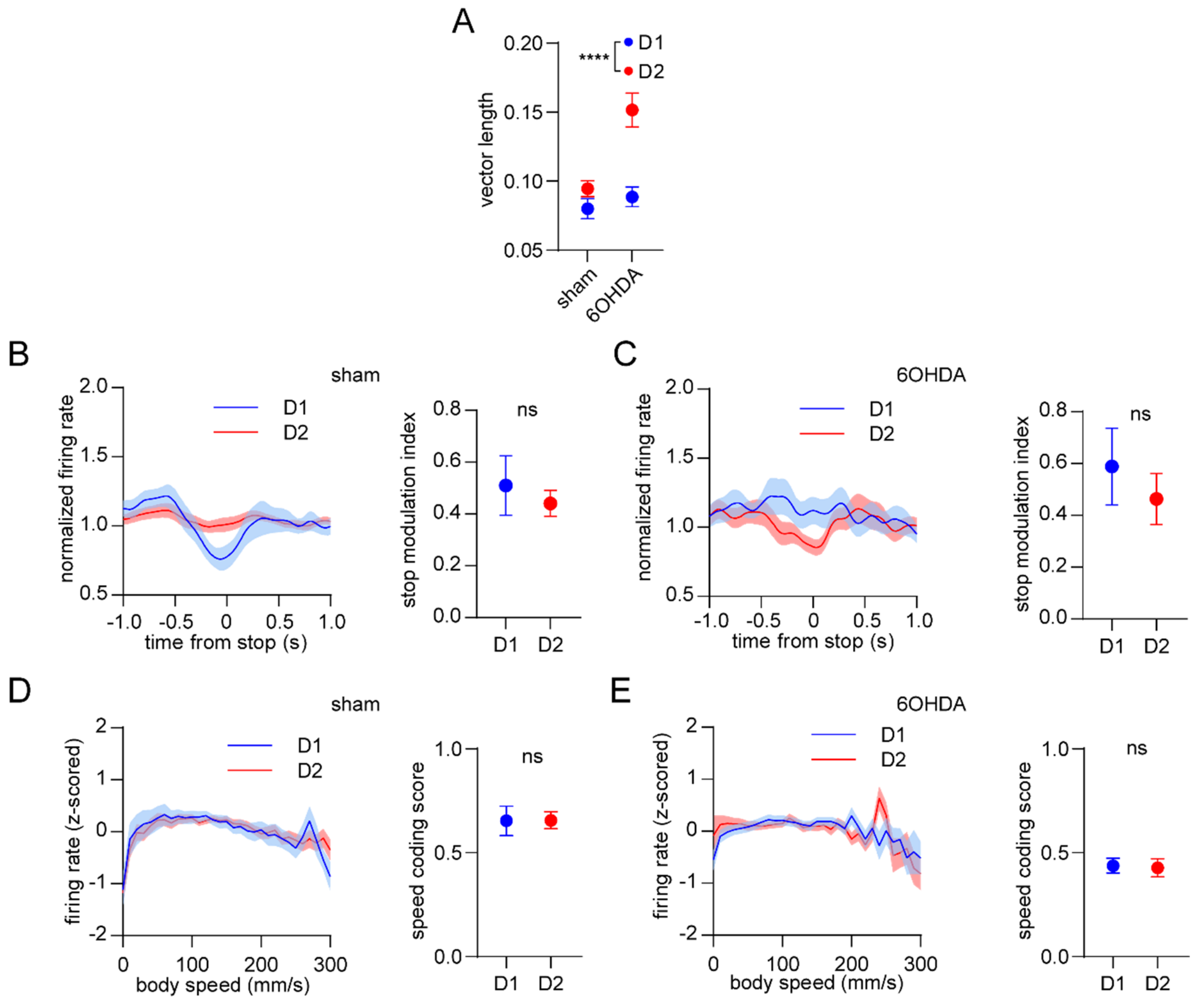

Figure S3. Dopamine lesions do not alter the balanced D1/D2 MSN encoding of movement cessation and body speed.

(A) Cell type (D1 or D2 MSN), group (sham or 6OHDA) and the interaction between them have a significant effect on the average vector length across the four limbs (n = 22 D1 and 57 D2 MSNs in the sham group, 31 D1 and 28 D2 MSNs in the 6OHDA group, two-way ANOVA, cell type factor: $F_{1,548} = 21$, $p < 0.0001$, group factor: $F_{1,548} = 15$, $p = 0.0001$, interaction: $F_{1,548} = 8$, $p = 0.004$).

(B) Left: normalized firing rate relative to the cessation of movement averaged across all D1 and D2 MSNs in the sham group. Data are normalized to the mean firing rate in a pre-stop period. Right: No significant difference in the stop modulation index (fractional change in firing in stop period relative to pre-stop) between D1 and D2 MSNs (n = 22 D1 and 57 D2 MSNs, unpaired t-test, p = 0.52).

(C) Left: normalized firing rate relative to the cessation of movement averaged across all D1 and D2 MSNs in the 6OHDA group. Data are normalized to the mean firing rate in a pre-stop period. Right: No significant difference in the stop modulation index between D1 and D2 MSNs (n = 31 D1 and 28 D2 MSNs, unpaired t-test, p = 0.5).

(D) Left: firing rate (z-scored) as a function of body speed averaged across all D1 and D2 MSNs in the sham group. Right: No significant difference in speed coding score (absolute Pearson r of firing rate in relation to speed) between D1 and D2 MSNs (n = 22 D1 and 57 D2 MSNs, unpaired t-test, p = 0.97).

bioRxiv preprint doi: https://doi.org/10.1101/2023.10.06.561216; this version posted October 9, 2023. The copyright holder for this preprint (which was not certified by peer review) is the author/funder, who has granted bioRxiv a license to display the preprint in perpetuity. It is made available under aCC-BY 4.0 International license.

(E) Left: firing rate (z-scored) as a function of body speed averaged across all D1 and D2 MSNs in the 6OHDA group. Right: No significant difference in speed coding score between D1 and D2 MSNs (n = 31 D1 and 28 D2 MSNs, unpaired t-test, p = 0.93). All data are represented as mean ± SEM.
